# Genetic Factors Regulating Lung Vasculature and Immune Cell Functions Associate with Resistance to Pneumococcal Infection

**DOI:** 10.1371/journal.pone.0089831

**Published:** 2014-03-03

**Authors:** Magda S. Jonczyk, Michelle Simon, Saumya Kumar, Vitor E. Fernandes, Nicolas Sylvius, Ann-Marie Mallon, Paul Denny, Peter W. Andrew

**Affiliations:** 1 Department of Infection Immunity and Inflammation, University of Leicester, Leicester, United Kingdom; 2 MRC Harwell, Mammalian Genetics Unit, Oxford, United Kingdom; 3 Department of Genetics, University of Leicester, Leicester, United Kingdom; Centers for Disease Control & Prevention, United States of America

## Abstract

*Streptococcus pneumoniae* is an important human pathogen responsible for high mortality and morbidity worldwide. The susceptibility to pneumococcal infections is controlled by as yet unknown genetic factors. To elucidate these factors could help to develop new medical treatments and tools to identify those most at risk. In recent years genome wide association studies (GWAS) in mice and humans have proved successful in identification of causal genes involved in many complex diseases for example diabetes, systemic lupus or cholesterol metabolism. In this study a GWAS approach was used to map genetic loci associated with susceptibility to pneumococcal infection in 26 inbred mouse strains. As a result four candidate QTLs were identified on chromosomes 7, 13, 18 and 19. Interestingly, the QTL on chromosome 7 was located within *S. pneumoniae* resistance QTL (*Spir1*) identified previously in a linkage study of BALB/cOlaHsd and CBA/CaOlaHsd F2 intercrosses. We showed that only a limited number of genes encoded within the QTLs carried phenotype-associated polymorphisms (22 genes out of several hundred located within the QTLs). These candidate genes are known to regulate TGFβ signalling, smooth muscle and immune cells functions. Interestingly, our pulmonary histopathology and gene expression data demonstrated, lung vasculature plays an important role in resistance to pneumococcal infection. Therefore we concluded that the cumulative effect of these candidate genes on vasculature and immune cells functions as contributory factors in the observed differences in susceptibility to pneumococcal infection. We also propose that TGFβ-mediated regulation of fibroblast differentiation plays an important role in development of invasive pneumococcal disease. Gene expression data submitted to the NCBI Gene Expression Omnibus Accession No: GSE49533

SNP data submitted to NCBI dbSNP Short Genetic Variation http://www.ncbi.nlm.nih.gov/projects/SNP/snp_viewTable.cgi?handle=MUSPNEUMONIA.

## Introduction

*Streptococcus pneumoniae* is an important pathogen of humans. It is estimated that pneumococcal diseases cause 5–35% mortality worldwide while less severe pneumococcal infections give rise to high costs of healthcare [1], [2]. Numerous reports show that infection, even with a highly virulent pneumococcal strain, may not result in death or severe clinical outcome in all infected individuals [3], [4], [5], [6], [7]. This observation suggests there is a genetic component to host susceptibility. However, although the pathogenesis of pneumococcal disease and determinants of pneumococcal virulence are quite well described [8], [9], [10], [11], [12], [13], [14], [15], little is known about the genetic aspects of host susceptibility. Identification of the host factors important in resistance or susceptibility to pneumococci could help develop new biomarkers, find novel drug targets and improve our knowledge about the disease and recovery.

Attempts to characterize the genetic factors in pneumococcal pneumonia susceptibility in humans have predominantly taken a candidate gene approach and been limited to a small number of pneumonia cases [16], [17], [18], [19], [20]. Unfortunately, the candidate gene approach precludes identification of new genes involved in the disease. Furthermore, while studying a human population a range of factors other than host genetic background can affect the power of disease associations. For example, misdiagnosis of the cases, as well as a number of confounding factors affecting the phenotype, such as previous exposures, lifestyle or medical treatment, can significantly increase noise in the data.

An alternative approach is to use mouse models of pneumococcal diseases. Presently, most of the data on host susceptibility in mice come from comparative studies of pairs of mouse strains representing opposite disease phenotypes (e.g.: resistant and susceptible as measured by animal survival). Such studies have focused on immune cell profiles and inflammatory responses (predominantly in lungs) at different stages of the disease [3], [6], [8], [9], [21], [22]. Although these studies delivered much useful information, they have no power to explain what genetic components are responsible for observed phenotypic differences.

Previously we undertook mapping of the disease susceptibility loci using BALB/c and CBA/Ca crosses and a *S. pneumoniae* infection resistance QTL 1 (*Spir1*) was identified on proximal chromosome 7 [23]. The locus spans a 7cM region encompassing too many genes to easily hypothesise a candidate.

A huge drawback in linkage analysis is its low mapping resolution that rarely leads to gene discovery. Additionally, a genetic effect of the QTL may be attributed to the combined effect of several physically linked genetic elements and can be disrupted during an attempt to narrow the QTL [24], [25], [26], [27], [28]. Therefore, linkage analysis can benefit hugely from Genome Wide Association studies (GWAS), which attains high mapping resolution [29], [30]. Recently GWAS studies in humans proved to be very successful in identification of causal genes involved in common diseases such as diabetes, HDL- and LDL-cholesterol regulation, systemic lupus or Crohn disease [31]. Nevertheless, the GWAS in humans remain very expensive, require tens of thousands of subjects and a huge bioinformatics effort [32]. In comparison, studies in mice can successfully identify disease associations using a fraction of these resources [24], [27]. They are also highly reproducible and benefit from availability of excellent mouse models for further functional characterisation of candidate genes [24], [33], [34].

Currently the Jackson Laboratory (JAX) holds the largest repository of mouse strains (JAX-mouse strains). It offers a wide range of inbred, mutant and genetically engineered mouse strains for which the dense genotyping data and other relevant information are freely available. Recently 17 of the inbred mouse strains from the Jackson Laboratory have been fully sequenced by the Sanger Institute [35]. All these advances in mouse genetics make the mouse increasingly attractive for the genetic dissection of complex traits.

In this study a panel of inbred JAX-mouse strains was evaluated for their susceptibility to infection with pneumococci. Collected phenotype data were then analysed by Efficient Mixed Model Association (EMMA) and Haplotype Association Mapping (HAM) to identify susceptibility loci. The complete sequences within the identified disease QTLs were then examined to narrow the number of candidate genes to those carrying the phenotype-specific polymorphism. Comparative analysis of the pulmonary gene expression in resistant and susceptible mouse strains was also used to support hypotheses of candidate genes and pathways. This approach identified three putative novel susceptibility QTLs, significantly narrowed the previously identified *Spir1* locus [23] and selected a small number of gene candidates for further hypotheses.

Our results indicate an important role of lung vasculature remodelling and immune cell regulation in the resistance to pneumococcal infection. We demonstrated that a prominent feature associated with the resistance was lung perivascular area free from cellular infiltrates. Subsequently we discussed the possible role of lung fibroblasts and/or smooth muscle cells in the resistance to pneumococcal infections. We also hypothesize a role of candidate genes in regulation of the TGFβ pathway and the vascular and immune cell functions in relation to the resistance to pneumococcal infection.

## Results

### Differences in susceptibility to pneumococcal infection in inbred mouse strains

Variation in susceptibility to pneumococcal infection was examined in 26 inbred JAX-mouse strains and the two Harlan strains, BALB/cOlaHsd and CBA/CaOlaHsd previously used in a linkage study of *S. pneumoniae* susceptibility [23]. Mice were infected intranasally with *S. pneumoniae* strain D39 and observed for 168 h (7 days), after which time each mouse that was alive was considered to have survived the infection. The shortest survival time was 21 h (LP/J) and the longest (for non-survivors) was 154 h (A/J) and the average survival time (excluding survivors) was 49 h. 32% of all tested animals survived infection but among the tested mouse strains the survival rate ranged from 100% to 0%. Survival differed significantly (p<0.001) between the mouse strains as measured by the time (hours) animal survived post-infection (Table 1). The susceptible mouse strains differed significantly in their survival from the resistant strains (p<0.0001) and there was a group of mouse strains, classified as intermediate, that differed from none of the tested strains or only from the strains representing the most extreme phenotype within the resistant or susceptible group of mice (p-value between 0.05–0.01). The resistant strains had a survival rate of 70%–100%, the susceptible 0%–20%, and the intermediate 30%–50%, (Table 1).

**Table 1:** Survival of tested inbred mouse strains and disease phenotype after intranasal infection with *S. pneumoniae*, D39.

Mouse strain	Mean survival (h)	SE	Survival rate (%)	Group
LP/J	31	3.4	0	susceptible
C3H/HeJ	31	3.7	0	susceptible
NZW/LacJ	40	1.9	0	susceptible
CBA/CaOlaHsd	46	13.7	10	susceptible
129S1/SvImJ	52	13.1	10	susceptible
CBA/J	54	4.1	0	susceptible
SJL/J	54	13.0	10	susceptible
SEA/GnJ	56	4.0	0	susceptible
BTBRT+ tf/J	59	11.7	0	susceptible
I/LnJ	59	4.9	0	susceptible
SM/J	69	17.5	20	susceptible
PL/J	75	16.1	20	susceptible
NOD/ShiLtJ	77	16.5	30	intermediate
C57BLKS/J	81	21.9	30	intermediate
DBA/1J	84	18.8	30	intermediate
C57L/J	87	17.8	30	intermediate
AKR/J	91	21.1	40	intermediate
DBA/2J	94	20.3	40	intermediate
C57BL/6J	95	20.0	40	intermediate
MA/MyJ	104	21.3	50	intermediate
C57BL/10J	116	18.7	50	intermediate
C58/J	119	16.9	50	intermediate
A/J	140	17.4	70	resistent
FVB/NJ	154	9.2	80	resistent
BALB/cJ	155	12.6	90	resistent
C57BR/cdJ	157	11.3	90	resistent
BALB/cOlaHsd	161	6.7	90	resistent
BALB/cByJ	168	0.0	100	resistent

SE – standard error.

**Figure 1:**
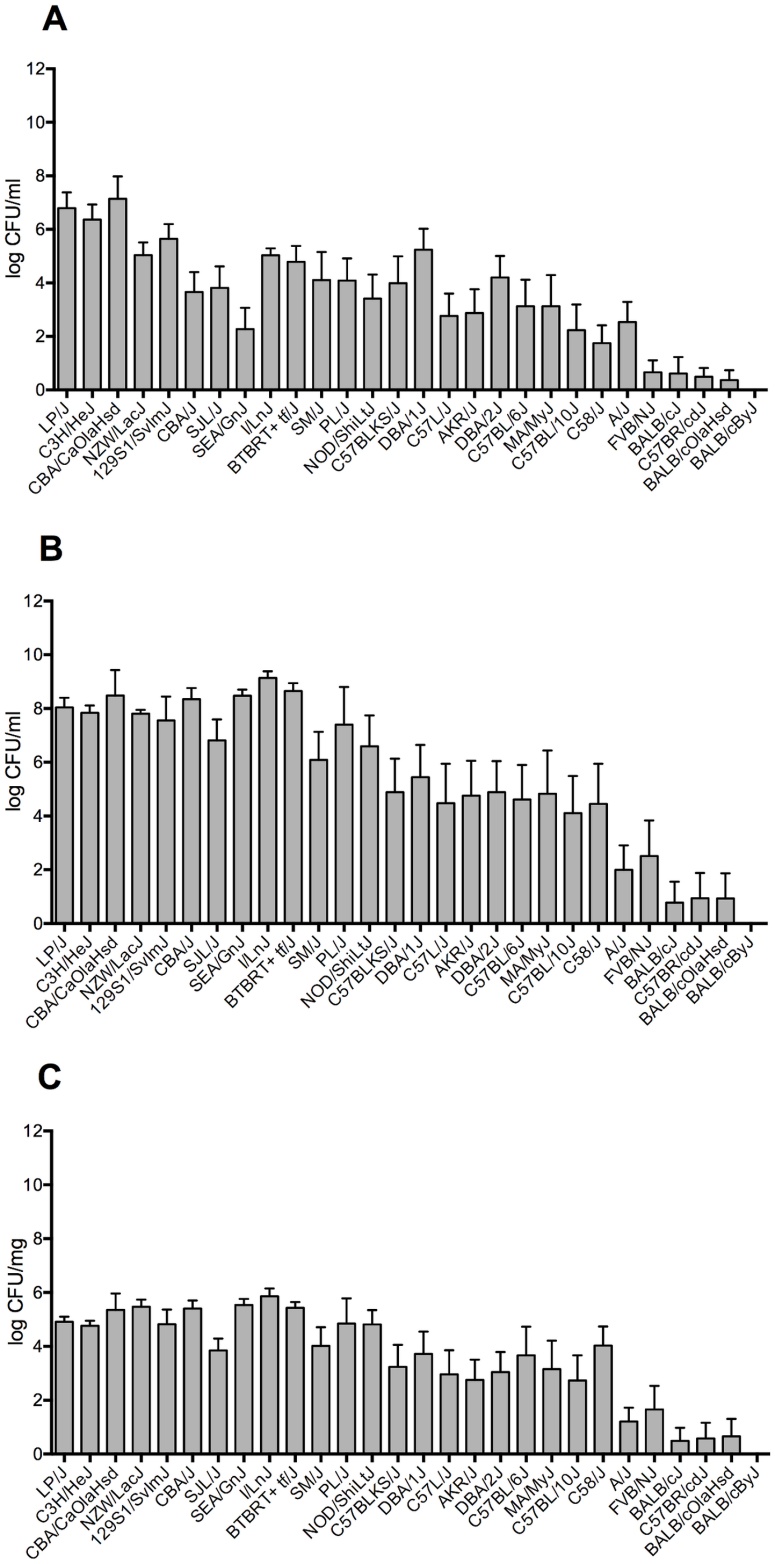
Bacterial counts in tissues of the tested inbred mice after intranasal-infection with *S. pneumoniae*, D39. Strains are ordered by increasing mean survival time post-infection. A) Bacterial count in blood 24 h post-infection B) Bacterial count in blood at the time of death C) Bacterial count in lungs at the time of death. Results are for a group of 10 animals. Error bar shows standard error (SE).

The bacterial load in tissues at the time of death was highly correlated with the survival time (h) with Pearson R^2^ = 0.83 and R^2^ = 0.87 for bacterial counts in lung and blood respectively. The level of bacteraemia at 24 h post-infection showed a lower correlation with the survival, with R^2^ = 0.7. Interestingly, we found that the intermediate strain, C58/J, with a 50% survival rate, had a very high bacterial load in the lungs at the time of death and all survivors had bacteria present in their lungs at the end of the experiment (Fig. 1C). Furthermore, the susceptible strain, SEA/GnJ, showed low bacterial counts in the blood at 24 h post infection, but nevertheless all animals died in less than 3 days. In contrast, the intermediate strains, DBA/1J and DBA/2J, despite the high 24 h bacteraemia survived on average 84 h and 94 h respectively, and had a 30%–40% survival rate (Fig. 1A, Table 1).

### Genetic susceptibility to *S. pneumoniae* infection is associated with mouse chromosomes 7, 13, 18 and 19

The survival time of the 26 inbred JAX-mouse strains were used to map candidate pneumonia susceptibility loci using two statistical approaches: the Efficient Mixed Model Associations (EMMA) and the Haplotype Association Mapping (HAM). The EMMA mapping was done using the EMMA server and the HAM mapping was done using PLINK software. Two SNP panels (132K and 4Mil) and data for individual mouse were used in the EMMA mapping. The P value was calculated for each SNP in the panels and the SNPs with a P value below 5×10^−8^ were considered significant. For the HAM model the 132K SNP panel and the average survival time were used and the P value cut-off for an inferred haplotype was 10^−5^. The QTL interval was defined as a region containing SNPs or haplotypes that passed the significance threshold plus 1 Mb upstream or downstream of this region.

Despite conceptual differences of the two methods, the results obtained with each were very similar (Table 2). The QTLs that passed the significance threshold in both mapping methods were selected for further analysis. Consequently four QTLs, on chromosomes 7, 13, 18 and 19, were identified. Interestingly the QTL on chromosome 7 identified in the GWAS approach was located within the previously reported *S. pneumoniae* infection resistance 1 QTL (*Spir1*) identified during linkage analysis of progeny of crosses between CBA/CaOlaHsd and BALB/cOlaHsd [23]. The Spir1 locus spans 7 cM and the GWAS analysis helped narrow this locus from 13 Mb (24 Mb–37 Mb) to 2.6 Mb (28.7 Mb–31.3 Mb).

**Table 2:** *S. pneumoniae* resistance QTLs identified by genome wide mapping from EMMA and HAM mapping using survival data (h).

QTL	Chromosome	interval (Mb)	(−)log(Pvalue) EMMA. 4Mil	(−)log(Pvalue) EMMA, 132K	(−)log(Pvalue) HAM
19:34 Mb	19	33.2–36.2	9.8	8.1	6.8
18:73 Mb	18	70.8–74.6	7.9	8.1	6.3
13:59 Mb	13	58–60.3	7.6	8.1	5.2
7:30 Mb	7	28.7–31.3	7.6	7.7	5.1

### The phenotype-associated SNPs were detected within 22 genes and intergenic regions

The SNPs that passed the genome-wide significance threshold (p<5×10^−8^) in the EMMA mapping, using the 4Mil SNP panel, were then extracted and the nucleotide variant at the selected SNP position was examined in the tested panel of 26 inbred JAX-mouse strains and in the two Harlan strains (CBA/CaOlaHsd and BALB/cOlaHsd). There were 17 significant SNPs within the QTL on chromosome 7, seven of them within coding sequences for *Ryr1*, *RasGRP4* and *Spint2* and two SNPs within regions annotated as regulatory by the Mouse Phenome Database (Table S1). Four significant SNPs were located within the QTL on chromosomes 13 (within *Ntrk2* gene) and five significant SNPs within the QTL on chromosome 18 (intergenic, upstream of *Smad4* gene) (Table S1). The QTL on chromosome 19 had the highest number of significant SNPs, 169, including 79 SNPs located within five proteins: *Stambpl1*, *Acta2*, *Fas*, *Slc16a12* and *Kif20b* (Table S1).

Comparison of the significant SNP variants between the tested mouse strains showed a clear separation of the resistant strains from the others (Table S1), although at some of the positions within the QTLs on chromosomes 18 and 19 a few intermediate mouse strains displayed a SNP variant associated with resistance (for example, C58/J, MA/MyJ and NOD/ShiLtJ within chromosome 18 and C57BLKS/J, C57L/J and C58/J within QTL on chromosome 19). Furthermore, in case of two resistant strains: A/J and C57BR/cdJ some of the of SNPs within the QTL on chromosome 7 displayed a susceptible variant. The later strain also displayed a susceptible SNP pattern within the QTL on chromosome 13 (Table S1).

A comprehensive analysis of the phenotype-associated SNP variation within the candidate QTLs was also done for the mouse strains for which the complete sequence data are available. Among these strains four represented resistant strains (BALB/cJ, A/J, FVB/NJ and BALB/cOlaHsd), five susceptible strains (LP/J, C3H/HeJ, 129S1/SvImJ, CBA/J and CBA/CaOlaHsd) and four strains with intermediate phenotypes (AKR/J, NOD/ShiLtJ, DBA/2J, and C57BL/6J). In this approach a SNP was classified as phenotype-associated if it was shared exclusively by all the mouse strains representing a given phenotype (i.e. resistant, susceptible or intermediate). Only SNPs differentiating resistant mouse strains from both susceptible and intermediate strains were identified in this analysis. No SNPs were found associated only with susceptible or only with an intermediate phenotype. The resistance phenotype-associated SNPs were located within the intergenic regions and the nine genes (*Ryr1*, *RasGRP4*, *Spint2*, *Ntrk2*, *Stambpl1*, *Acta2*, *Fas*, *Slc16a12* and *Kif20b*) previously identified by EMMA and described above, but phenotype-associated SNPs also were found in a further 13 genes (Table 3). However, these newly identified 13 genes displayed a lower number of phenotype-associated SNPs than the genes identified during the EMMA mapping (Table 3).

**Table 3:** Phenotype-specific genetic polymorphisms within *S. pneumoniae* resistance QTLs.

No of SNPs	chrom	gene	consequence	EMMA
>400	7	*Ryr1*	non-synon, synon, splicing, intron	Y
100–300	19	*Stambpl1*	non-synon, synon, splicing, intron	Y
100–300	19	*Slc16a12*	non-synon, synon, splicing, intron	Y
100–300	19	*Fas*	non-synon, synon, intron	Y
100–300	13	*Ntrk2*	synon, intron	Y
10–100	19	*Acta2*	synon, intron	Y
10–100	19	*Ifit1*	non-synon, synon, intron	N
10–100	19	*Kif20b*	non-synon, synon, splicing, intron	Y
10–100	19	*Pank1*	synon, intron	N
10–100	7	*Kcnk6*	synon, intron	N
10–100	7	*Catsperg1*	synon, intron	N
10–100	7	*Map4k1*	non-synon, synon, intron	N
10–100	7	*Rasgrp4*	non-synon, intron	Y
10–100	7	*Spint2*	intron	Y
>10	13	*Agtpbp1*	intron	N
>10	7	*Yif1b*	intron	N
>10	7	*IMUP-1*	non-synon, intron	N
>10	7	*Psmd8*	synon	N
>10	7	*Spred3*	intron	N
>10	7	*Sipa1*	intron	N
>10	7	*Ppp1r14a*	intron	N

The number of phenotype-associated polymorphisms within the coding region of the QTLs and their consequences. The presented number of SNPs includes the SNPs identified in the EMMA mapping and in the analyses of the full-length sequences within the disease QTLs. No of SNPs– number of phenotype-associated SNPs identified within the gene; Chrom – chromosome, non-synon – SNP causing amino acid change, synon – SNP causing silent mutation, intron – SNP within intron, splicing – SNP within splicing site of the gene. EMMA – indicates whether the genome-wide significant SNPs (p-value< 5×10^−8^) were identified within the gene during the EMMA mapping: Y-yes, N-no.

The largest number of phenotype-associated SNPs was found within the *Ryr1* gene (over 400), including SNPs causing amino acid change or substitutions within a splicing site (Table 4). The highest number of phenotype-associated amino acid changes was found within the *Map4k1* sequence. There were a further eight genes identified with phenotype-associated amino acid changes (Table 4). The PROVEAN score for each of the detected non-synonymous alterations did not indicate any highly deleterious effect for the tested proteins, with the lowest PROVEAN score of −0.87 and −0.74 for RasGRP4 and FAS respectively (Table 4). Interestingly, the QTL on chromosome 18 was the only one for which the phenotype-associated SNPs were found exclusively within an intergenic region, namely between the *Dcc* and *Smad4* genes. Two more members of the SMAD family were found to be encoded by genes near this QTL: *Smad2* and *Smad7*. An additional SMAD gene, *Smad5*, was found located near the QTL on chromosome 13.

**Table 4:** Phenotype-specific genetic polymorphisms within *S. pneumoniae* resistance QTLs leading to amino acid change or alteration within the splicing site.

		Amino acid change			Splicing site	
*Gene name*	*Chrom*	*SNP_ID*	*change: R>S*	*PROVEAN score*	*SNP_ID or position*	*nt change: R>S*
*Map4k1*	7	rs31155356	R>C	0.329		
		rs31836292	I>V	−0.328		
		rs31836292	I>V	−0.328		
*Ryr1*	7	rs51295578	S>A	−0.383	29832579	A>G
					29842146	T>G
					rs48602222	G>A
*Stambpl1*	19	rs30934349	M>I	0.156	rs30754595	C>T
		rs30525805	N>S	0.04		
*Slc16a12*	19	rs46173389	T>C	−0.011	34749291	C>A
*Fas*	19	rs30844760	H>R	−0.738		
*Ifit1*	19	rs50377880	V>I	0.387		
*Kif20b*	19	rs13483601	E>G	1.586	rs30751713	A>G
*Rasgrp4*	7	rs45673726	I>M	−0.872		
*IMUP-1*	7	rs32347251	K>M	2.833		

change: R>S – amino acid or nucleotide change, resistant (R) *versus* susceptible (S) variant.

### The pulmonary transcriptome differed between resistant and susceptible mouse strains during pneumococcal infection

Differences in pulmonary gene expression between highly resistant (BALB/cOlaHsd) and highly susceptible (CBA/CaOlaHsd) mouse strains, during the early stage of pneumococcal infection (6 h post-infection), were then investigated. Gene expression was measured using an Illumina MouseWG-6_V2_0_R3 microarray and result submitted to the NCBI Gene Expression Omnibus, Accession No: GSE49533. The results were then validated by the real time RT-PCR of 15 selected genes. On average, an 81% correlation was observed between the two methods (Table S2). Genes were considered differentially expressed (DEG) if their expression changed at least 50% compared to sham-infected controls and there was a P value of <0.05. As a result, three lists of DEGs were created: BALB/cOlaHsd infected *versus* control, CBA/CaOlaHsd infected *versus* control and control BALB/cOlaHsd *versus* control CBA/CaOlaHsd. All three lists of DEGs were compared with the list of genes encoded within the QTLs. 30 genes located within the QTLs were differentially expressed in at least at one of the comparisons (Table 5). Five genes with detected phenotype-specific polymorphism were also among the DEGs (*Map4k1*, *Spint2*, *Fas*, *Ppp1r14a* and *Pank1*). Interestingly, *Smad2* located near to the QTL on chromosome 18 was differentially regulated in BALB/cOlaHsd, but not in CBA/CaOlaHsd, during the infection. Two additional members of the SMAD family: *Smad1* and *Smad6* were also differentially expressed during the disease in BALB/cOlaHsd and CBA/CaOlaHsd, respectively (Table 5).

**Table 5:** Differentially expressed genes (DEGs) within candidate susceptibility QTL between mice intranasally infected with *S. pneumoniae*, D39 and control animals.

Chrom	QTL	QTL interval (Mb)	Gene name	BALB/c infection	CBA/Ca infection	BALB/CBA controls	BALB/CBA infection
7	7:30 Mb	28.7–31.3	Dmkn	1.6	2.4		−2.4
7	7:30 Mb	28.7–31.3	Ffar2	3.1	4.4		
7	7:30 Mb	28.7–31.3	Fxyd3	1.6		−1.9	
7	7:30 Mb	28.7–31.3	Hcst		1.6	1.6	
7	7:30 Mb	28.7–31.3	Nfkbib	2			
7	7:30 Mb	28.7–31.3	Nfkbid	2.8	3.3		
7	7:30 Mb	28.7–31.3	Ppp1r14a	−1.5			1.8
7	7:30 Mb	28.7–31.3	Tyrobp	1.8	1.8		
7	7:30 Mb	28.7–31.3	Zfp260	−1.5		2.2	1.5
13	13:59 Mb	58–60.3	Isca1		−1.5		
18	18:74 Mb	70.8–74.6	Mbd1	3.1	2.9	−1.9	
19	19:34 Mb	33.2–36.2	Ankrd1	2	1.9	−2.3	−2.5
19	19:34 Mb	33.2–36.2	Ch25h	7.2	5.7	−1.5	−1.7
19	19:34 Mb	33.2–36.2	Fas	2.8	2.3	−1.5	
19	19:34 Mb	33.2–36.2	Ifit3		−1.5	−1.6	−1.6
19	19:34 Mb	33.2–36.2	Pank1	−1.8	−1.6	−2.5	
7	7:30 Mb	28.7–31.3	Capns1			−1.9	
7	7:30 Mb	28.7–31.3	Cd22			5.9	5.2
7	7:30 Mb	28.7–31.3	Cox7a1			2	
7	7:30 Mb	28.7–31.3	Lsr			−2.2	
7	7:30 Mb	28.7–31.3	Map4k1			1.8	
7	7:30 Mb	28.7–31.3	Mrps12			−2.9	−3.2
7	7:30 Mb	28.7–31.3	Psmd8			−10.5	−8.1
7	7:30 Mb	28.7–31.3	Spint2			−1.6	
7	7:30 Mb	28.7–31.3	Zbtb32			2.6	3.1
7	7:30 Mb	28.7–31.3	Zfp27			−2.4	−2
7	7:30 Mb	28.7–31.3	Zfp30			2.5	2.5
7	7:30 Mb	28.7–31.3	Rasgrp4				−2.5
7	7:30 Mb	28.7–31.3	Hspb6				−1.6
7	7:30 Mb	28.7–31.3	Zfp383				−2
13	13:59 Mb	58–60.3	Ubqln1			−1.9	
18	18:74 Mb	70.8–74.6	Elac1			2.2	2.1
18	18:74 Mb	70.8–74.6	Me2			−3	−2.2
18	18:74 Mb	70.8–74.6	Smad2	−1.5		−1.8	−3
8	N/A	N/A	Smad1	1.8			
9	N/A	N/A	Smad6		−1.8		

Pulmonary gene expression 6 h post-infection is represented as fold change between infected and non-infected animals (BALB/c infection: BALB/c-infected *versus* PBS-treated, CBA/Ca infection: CBA/Ca infected *versus* PBS-treated), between PBS-treated control animals (BALB/CBA: BALB/c *versus* CBA/Ca PBS-treated) and between infected BALB/c and infected CBA/Ca (BALB/CBA infection). Chrom. - chromosome.

**Figure 2:**
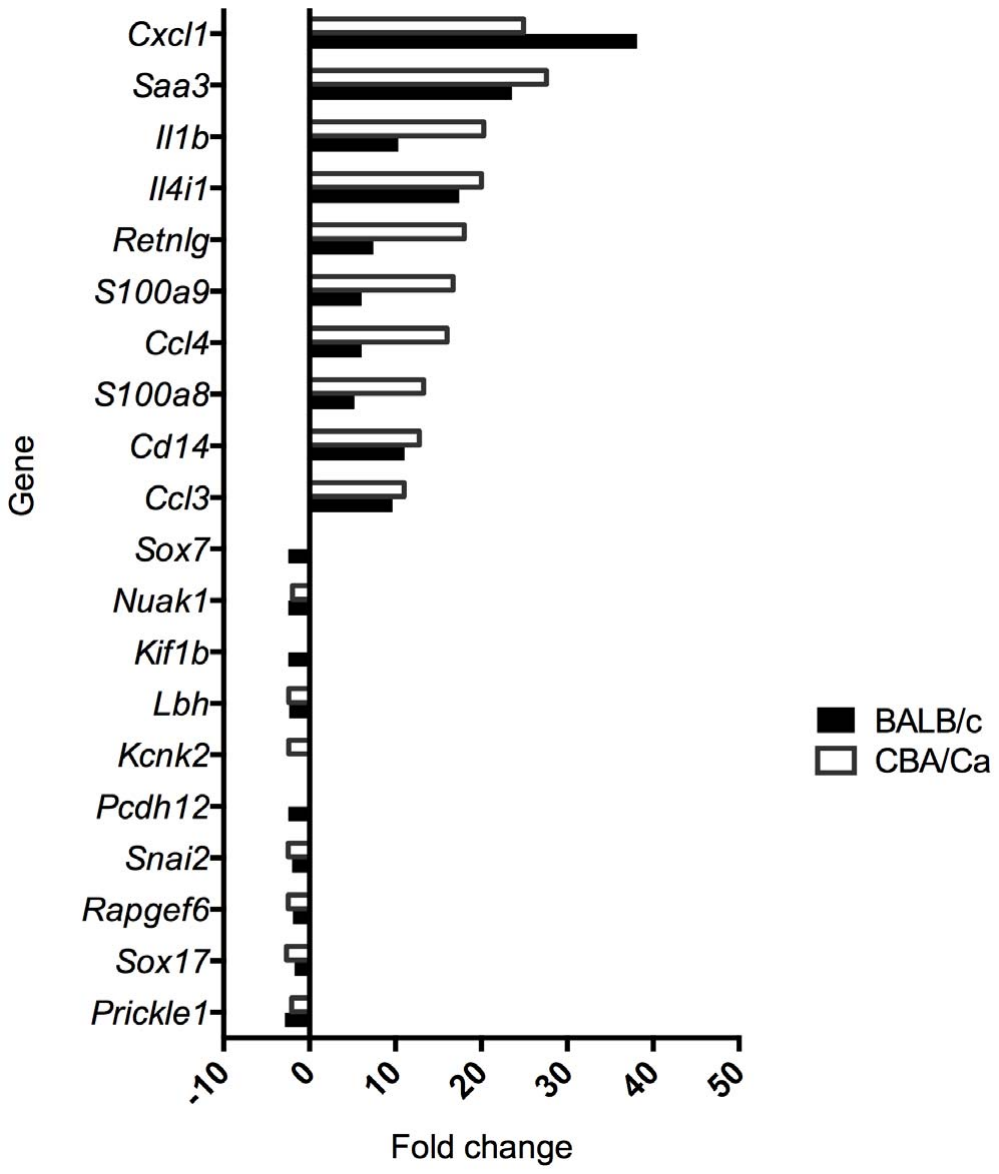
Change in gene expression 6-infection of BALB/cOlaHsd (resistant) and CBA/CaOlaHsd (susceptible) with *S. pneumoniae*, D39. Fold change is represented as compared to control animals (PBS-treated). Only the ten most up-regulated and ten most down-regulated genes are presented.

**Figure 3:**
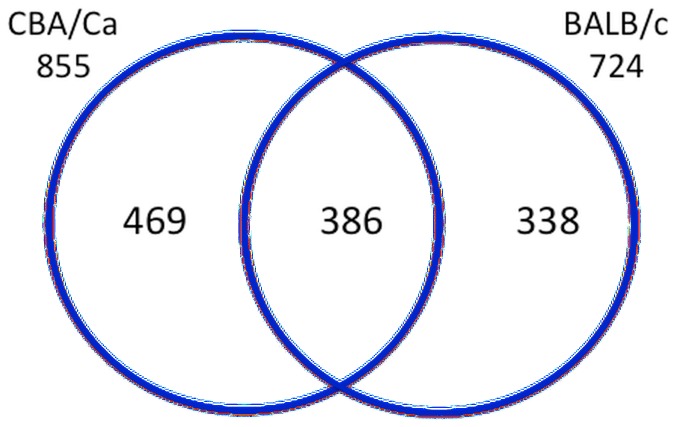
Number of differentially regulated genes (DEGs) during infection with *S. pneumoniae*, D39. The included genes show fold change in expression ≥1.5 and change is significant at p≤0.05. The total number of BALB/cOlaHsd (resistant) and CBA/CaOlaHsd (susceptible) DEGs is indicated below the strain number and amount of commonly and uniquely regulated genes is indicated within the Venn diagram.

The magnitude of DEGs up-regulation was much higher (about 40-fold change) than that of down-regulation (about 3-fold change) (Fig. 2). Approximately a half of the DEGs were found to be uniquely associated with the infection of only one of the tested mouse strains, either BALB/cOlaHsd or CBA/CaOlaHsd (Fig. 3). Interestingly, though, the magnitude of change of these strain-specific DEGs (uDEGs) was relatively low (up to 4-fold) (Table 6). If the ratio of expression in the two mouse strains of the most highly up-regulated genes (above 4-fold change during infection) was calculated BALB/cOlaHsd and CBA/CaOlaHsd differed at least 2-fold in the regulation of genes encoding leukocytes chemoattractants and protease inhibitors (Table 7).

**Table 6:** Differentially expressed genes unique (uDEGs) in either BALB/cOlaHsd (resistant) or CBA/CaOlaHsd (susceptible) after intranasal-infection with *S. pneumoniae*, D39.

	BALB/c		CBA/Ca	
*uDEG*	*FOLD*	*PVALUE*	*FOLD*	*PVALUE*
*Egr1*	4.1	1.00E-04		
*Plek*	3.0	8.10E-03		
*Srgn*	3.0	1.90E-03		
*Nol5*			2.9	4.00E-04
*Fos*	2.7	1.00E-04		
*Ptpn22*	2.6	1.00E-04		
*Cyp7b1*			2.5	1.90E-03
*Iigp2*			2.4	1.40E-03
*Abcb1b*			2.4	1.60E-03
*Igtp*			2.3	7.30E-03
*Kif1b*	−2.0	8.00E-04		
*Bcl9l*	−2.1	1.00E-04		
*Ehd4*			−2.1	1.75E-02
*Nrp*	−2.2	1.36E-02		
*Cidec*			−2.2	3.60E-03
*Gdf10*			−2.2	9.00E-04
*Krtap17-1*			−2.3	3.00E-04
*Sox7*	−2.4	1.00E-04		
*Kif1b*	−2.4	1.00E-04		
*Kcnk2*			−2.4	1.50E-03

Expression is represented as fold change (infected animals versus PBS-treated). Only the five most up-regulated and the five most down-regulated uDEGs are presented for each strain.

**Table 7:** Differentially expressed genes during intranasal-infection with *S. pneumoniae*, D39 in both BALB/cOlaHsd and CBA/CaOlaHsd and showing at least two-fold change difference between the two strains during the infection.

DEG	BALB/c infection	CBA/Ca infection	Fold difference CBA/BALB infected
*Serpina3g*	2.4	7.7	3.2
*Serpina3f*	3.1	9.8	3.1
*S100a9*	6.0	16.7	2.8
*Ccl4*	6.0	16.0	2.6
*S100a8*	5.2	13.3	2.6
*Retnlg*	7.4	18.0	2.4
*Fcgr2b*	2.7	5.4	2.0
*Il1b*	10.3	20.3	2.0
*F13a1*	2.4	4.7	1.9
*Cdkn1a*	4.1	2.2	−1.9
*Fkbp5*	5.6	2.7	−2.1

The DEGs also were analysed with the web-based DAVID tool, in order to identify pathways in which DEGs were over-represented. Consequently, pathways involved in cell proliferation and differentiation (hematopoietic lineage pathway and p53-signalling) and axon guidance signalling were uniquely associated with the pulmonary transcriptome of the resistant BALB/cOlaHsd mice during the infection (Table 8). Interestingly the hematopoietic pathway was also among the most significant pathways when DEGs of control animals (BALB/cOlaHsd *versus* CBA/CaOlaHsd PBS-treated) were compared (Table 8). The pathways unique for susceptible mice were adipocytokine signalling, natural killer cell mediated cytotoxicity, T-cell receptor signalling and cytosolic DNA-sensing pathway (Table 8). Interestingly majority of these pathways are related to immune cell function. Furthermore, significant enrichment of genes involved in vasculature remodelling and responses to wounding was found in both mouse strains during the infection. The enrichment score for these two clusters was between 8.3 and 10.6, while enrichment scores of any subsequent functional clusters were below 5.5 (Table 9). There was no functional cluster with enrichment score 5 or more for the pulmonary transcriptome of control animals.

**Table 8:** Significantly regulated pathways (KEGG) during infection and differentiating PBS-treated BALB/c and CBA/Ca mice.

KEGG pathway	BALB/c	CBA/Ca	BALB/CBA control
	*PValue*	*PValue*	*PValue*
Cytokine-cytokine receptor interaction	3.92E-07	2.38E-08	
Pathways in cancer	2.92E-05	6.33E-07	
Toll-like receptor signalling pathway	3.32E-04	1.33E-06	
B cell receptor signalling pathway	3.10E-03	4.43E-05	
Chemokine signalling pathway	9.02E-03	5.10E-05	
MAPK signalling pathway	1.93E-05	1.08E-04	
Apoptosis	1.57E-03	1.09E-04	
Jak-STAT signalling pathway	1.88E-04	3.02E-03	
NOD-like receptor signalling pathway	8.63E-03	5.88E-03	
Hematopoietic cell lineage	1.20E-03		4.30E-04
Axon guidance	3.92E-03		
p53 signalling pathway	4.33E-03		
Adipocytokine signalling pathway		1.56E-04	
Natural killer cell mediated cytotoxicity		9.83E-04	
T cell receptor signalling pathway		6.06E-03	
Cytosolic DNA-sensing pathway		1.05E-02	
Antigen processing and presentation			8.79E-05
Allograft rejection			5.73E-04
Autoimmune thyroid disease			1.10E-03
Graft-versus-host disease			2.16E-03
Type I diabetes mellitus			4.05E-03
Cell adhesion molecules (CAMs)			4.95E-03
Systemic lupus erythematosus			8.53E-03

Names of pathways (KEGG) and their significance (PValue) are provided.

**Table 9:** Functional annotation clustering of differentially expressed genes during pneumococcal infection of BALB/cOlaHsd and CBA/CaOlaHsd.

		BALB/c	CBA/Ca
*ID*	*Description*	*PValue*	*PValue*
GO:0001944	vasculature development	1.50E-11	3.89E-10
GO:0001568	blood vessel development	3.66E-11	2.04E-10
GO:0048514	blood vessel morphogenesis	5.34E-10	1.78E-09
GO:0001525	angiogenesis	1.79E-08	1.36E-07
	**Annotation Cluster 1 Enrichment Score:**	**9.6**	**8.7**
GO:0009611	response to wounding	1.71E-09	1.67E-12
GO:0006954	inflammatory response	1.01E-10	4.72E-12
	inflammatory response	1.06E-08	2.20E-10
GO:0006952	defense response	4.12E-07	2.35E-10
	**Annotation Cluster 2 Enrichment Score:**	**8.3**	**10.6**
GO:0042035	regulation of cytokine biosynthetic process		6.54E-07
GO:0042108	positive regulation of cytokine biosynthetic process		2.81E-06
GO:0043123	positive regulation of I-kappaB kinase/NF-kappaB cascade		2.46E-05
	**Annotation Cluster 3 Enrichment Score:**		**5.4**

Only clusters with enrichment score above 5 are shown. Information provided: the overall enrichment score for the cluster based on EASE score of each member (Enrichment score), name of annotation (Description) and its GO code (ID) if applicable, p-value for the term (PValue).

### Lung histopathology of BALB/cOlaHsd and CBA/CaOlaHsd mice showed differences in immune cells recruitment

**Figure 4:**
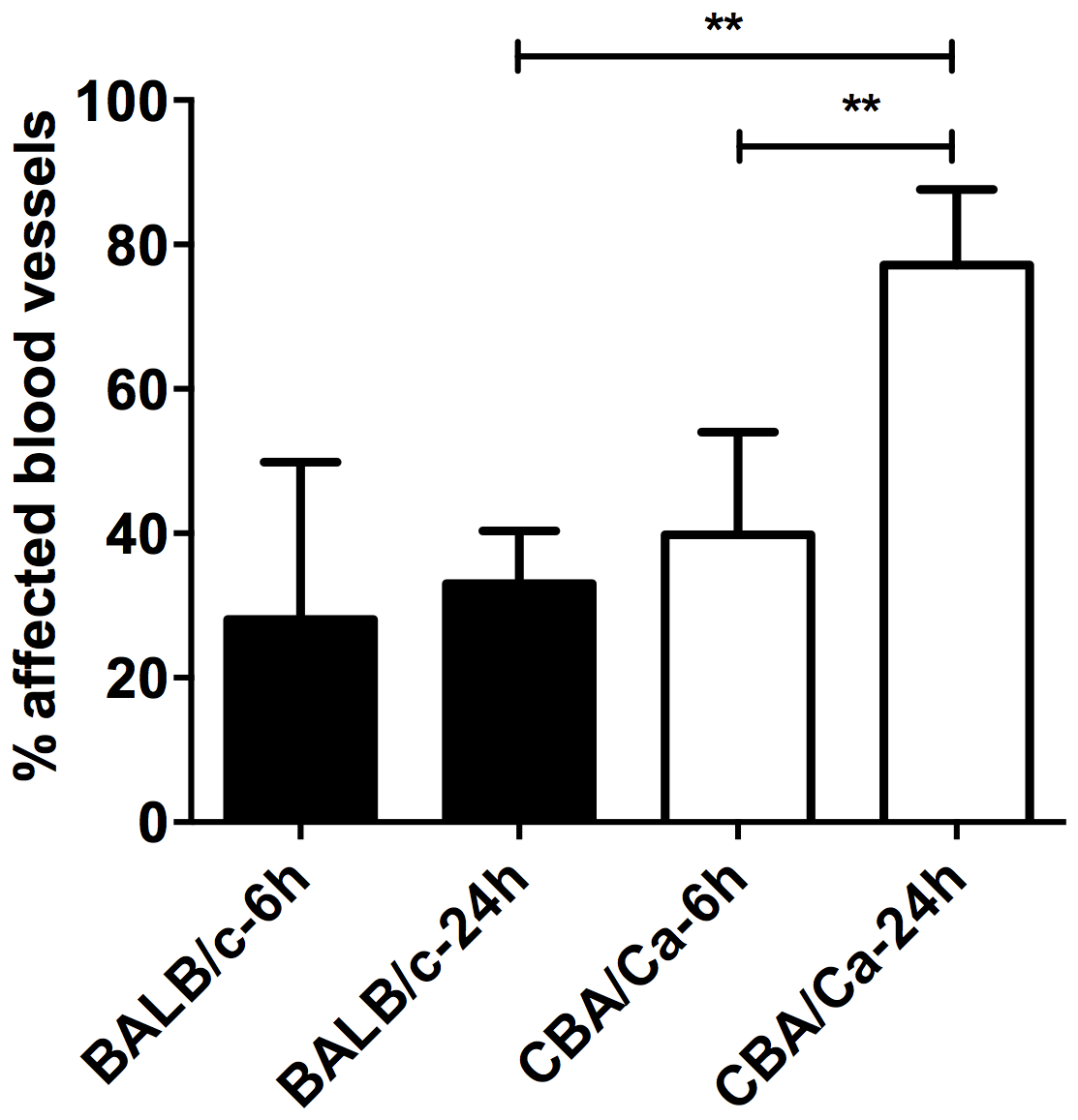
Percentage of lung's blood vessel affected by inflammation within perivascular areas after infection with *S. pneumoniae* D39. Results are presented for 6-infection for BALB/cOlaHsd (black bars) and CBA/CaOlaHsd (open bars), from two independent experiments, with a total of 5 animals per group. ** p<0.001.

**Figure 5:**
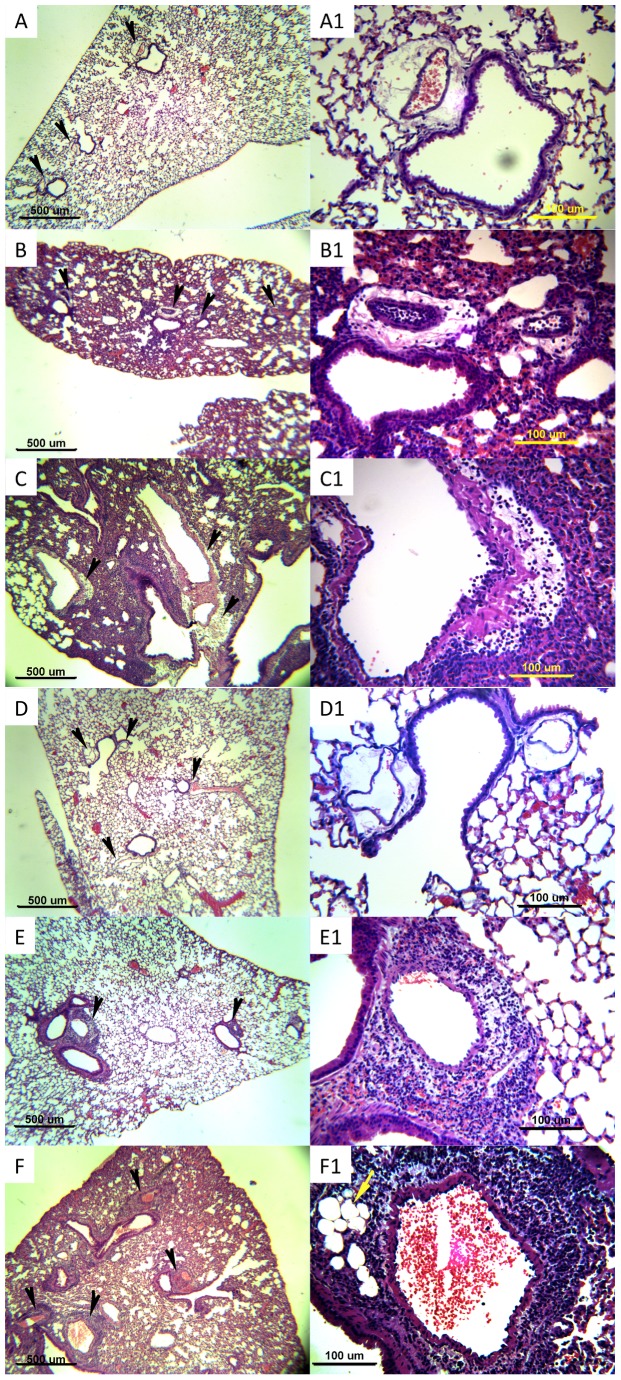
Inflammation pattern in lungs of BALB/cOlaHsd and CBA/CaOlaHsd mice after infection with *S. pneumoniae*, D39. Hematoxylin-and-eosin-stained paraffin wax lung sections from BALB/cOlaHsd (A, A1–C,C1) and CBA/CaOlaHsd (D,D1–F,F1). Panels A,A1 and D,D1 represent lungs of PBS-treated animals, panels B,B1–C,C1 and E,E1–F,F1 are lung sections at 24 h-post infection. Panels A–F represent magnification 40× and panels A1–F1 magnification 200×. Bar indicates 500 µm for 40× magnification and 100 µm for 200× magnification. Black arrows indicate perivascular areas. Yellow arrow in panel F1 indicates perivascular adipose tissue.

Examination of lung histopathology showed that about 30% of perivascular areas of resistant BALB/cOlaHsd and almost 80% of susceptible CBA/CaOlaHsd mice were affected by cellular infiltration at 24 h post-infection and this difference was statistically significant. However the difference between both mouse strains was not observed at 6 h post-infection when about 30%–40% of perivascular areas of both CBA/CaOlaHsd and BALB/cOlaHsd were affected by cellular infiltrates (Fig. 4). Interestingly, the perivascular areas of resistant BALB/cOlaHsd mice were largely unaffected by immune cells infiltration even though considerable inflammation was present within the lung parenchyma. In contrast, the susceptible CBA/CaOlaHsd mice had substantial accumulation of immune cells within perivascular areas, even when no visible infiltration was observed within alveolar space (Fig. 5).

## Discussion

Pneumococcal infections can lead to a high mortality and morbidity in humans. While substantial effort has been made to understand the pneumococcal attributes that explain this situation, almost nothing has been done to explain the fact that individuals vary in their innate susceptibility to these infections. Identification of the host factors explaining innate resistance or susceptibility would help development of alternative, host-directed therapies and screening tests to identify individuals at risk. In the present study we aimed to identify host factors determining resistance to pneumococcal infection, using mouse models of pneumonia and bacteraemia. We phenotyped a panel of genetically defined inbred mouse strains for their resistance to pneumococcal infection and looked for association between the host genetic background and disease phenotype. Furthermore we analysed differences in gene expression and lung pathology between two mouse strains representing opposite disease phenotypes (susceptible CBA/CaOlaHsd versus resistant BALB/cOlaHsd).

The tested panel of inbred mouse strains displayed variable disease phenotypes, ranging from death to complete resistance. The time that animal survived the infection (survival) was used to map pneumococcal pneumonia susceptibility/resistance loci and four candidate QTLs on chromosomes 7, 13, 18 and 19 were identified. It is noteworthy that the candidate QTL on chromosome 7 is located within the *S. pneumoniae* resistance QTL 1 (*Spir1*), identified by our previous linkage analysis of BALB/cOlaHsd (refer thereafter as BALB/c) and CBA/CaOlaHsd crosses (refer thereafter as CBA/Ca) [23]. Subsequently, the GWAS mapping helped to narrow down the *Spir1* locus from 13 Mb to 2.6 Mb. The most statistically significant SNPs identified by EMMA were located within nine genes, or in intergenic regions, and were able to differentiate the resistant from the susceptible mouse strains, therefore they were regarded as phenotype-specific (or phenotype-associated).

We took advantage of the availability of the full nucleotide sequences of thirteen of the tested inbred stains [35] to search for the genes or intergenic regions, carrying potential causal variants. This approach was possible because within the subset of the sequenced inbred strains, the resistant, susceptible and intermediate disease phenotypes were matched in numbers. The analysis resulted in identification of further phenotype-specific SNPs within the nine genes selected by the association analysis, but additionally found thirteen other candidate genes within the QTLs. Among these candidate genes was a member of a solute carrier transporter family: *Slc16a12*. A large number of phenotype-associated SNPs were identified within this gene, some of them leading to a change within the amino acid sequence and the splicing site. This is worth mentioning because one of the best-known genetic factors influencing susceptibility to bacterial infection is a solute carrier transporter, *Slc11a1* (also known as *Nramp1*) [36], [37]. The *Slc11a1* gene was not associated with pneumococcal disease in our GWAS but finding another member of the same family associated with resistance to pneumococcal infection may suggest a broader role of the solute carrier transporters during bacterial infection. The transporter associated with pneumococcal disease, *Slc16a12*, belongs to the monocarboxylate transporters [38]. Monocarboxylates, such as lactate, pyruvate or ketones, are important elements of several metabolic pathways and therefore their availability to pneumococci could be crucial for bacterial survival and virulence within the host.

However, the susceptibility to complex diseases such as infectious diseases, most likely depends on networks of genes, with additive genetic effects, or on groups of proteins forming interacting networks [24], [28]. Indeed, previous reports show that altered genes underlying disease susceptibility were frequently clustered within the same or related pathways [39], [40]. Therefore the discovery of multiple candidate genes and QTLs involved in the susceptibility to pneumococcal pneumonia initiated our investigation into common aspects of the candidate genes, such as signalling pathways, functional similarities or tissue-specificity.

The presence of a cluster of genes encoding members of the SMAD family within or nearby two of the disease QTLs (*Smad4*, *Smad2* and *Smad7* on chromosome 18 and *Smad5* on chromosome 13) focused our attention on the TGFβ pathway, in which SMAD proteins are key factors [41], [42]. As well as the SMAD genes we found two other candidate genes with extensive phenotype-specific polymorphism that are associated with the TGFβ pathway: *Stambpl1* and *Acta2* (both within the QTL on chromosome 19). The former gene encodes the STAM binding protein-like 1 that interacts with SMAD2 and SMAD7 [43] and the later gene encodes actin smooth muscle, a target gene of TGFβ signalling [44], [45].

Gene expression analysis showed that *Smad2* is significantly down-regulated while *Smad1* and the activin receptor gene *Acvr1b* [43] are up-regulated in the resistant BALB/c mouse during infection, but not in the susceptible CBA/Ca. Interestingly, the *Smad6* and the TGFβ superfamily receptor *Acvrl1* [46] were significantly down-regulated in susceptible CBA/Ca but not in resistant BALB/c. These observations are in agreement with our previous results that demonstrated an important role of TGFβ in resistance to pneumococcal infection [21]. We reported significant differences in the concentrations of TGFβ in BALB/c and CBA/Ca mice and that the higher levels of TGFβ found in BALB/c lungs during pneumococcal infection correlated with the increase in the number of T regulatory cells (Treg) in the lungs. Furthermore we showed that blockade of TGFβ with a synthetic peptide blocked the induction of Treg cells and decreased BALB/c resistance to infection [21].

In addition to influencing certain immune cells, the TGFβ-pathway also regulates wound healing, angiogenesis and extracellular matrix production via activation of fibroblast differentiation [44]. The results of our study suggested fibroblast activation and functioning might play a role in the resistance to pneumococcal pneumonia. We demonstrated that the marker of fibroblast activation, *Acta2*, has extensive phenotype-associated polymorphism. Furthermore, we found that during pneumococcal infection the expression of genes known to regulate transcription of *Acta2* differed significantly between resistant and susceptible mice. For example, the candidate gene *Ch25h* encoded within the QTL on chromosome 19 is known to enhance *Acta2* expression and increase TGFβ1 release [47]. We showed that transcription of *Ch25h* significantly differed between resistant BALB/c and susceptible CBA/Ca during pneumococcal infection and between control animals. Interestingly, *Ch25h* is located near to the cluster of highly significant intergenic SNPs identified by the EMMA. Therefore it would be interesting to know whether the identified polymorphisms within this intergenic region are responsible for observed differences in *Ch25h* expression. Another example of an *Acta2*-regulating gene was the early growth response factor 1 (*Egr1*), up-regulated only by BALB/c during pneumococcal infection. *Egr1* is known to inhibit TGFβ-mediated *Acta2* expression [48].

Fibroblasts are ubiquitous cells, which in normal lungs residue in vascular adventitia and airway wall tissue. Beside their vital role in maintaining tissue structure and integrity, fibroblasts also regulate immune cells trafficking, site-specific accumulation and survival [49], [50], [51]. Furthermore the phenotypic differences between fibroblasts collected from different tissues or even different locations within the organ are widely reported [52], [53], [54]. One example of such heterogeneity is human pulmonary fibroblasts [49]. It was demonstrated that the fibroblasts residing in the bronchi show higher expression of extracellular matrix (ECM) proteins while the fibroblasts from lung parenchyma display increased TGFβ signalling and *Acta2* expression. The parenchymal fibroblasts, but not the bronchial ones can rapidly activate endogenous TGFβ via tension-mediated mechanism [54], [55], [56]. In the light of the above observations it is particularly interesting to see that the pattern of lung inflammation differs between resistant BALB/c and susceptible CBA/Ca. The cellular infiltrates are located predominantly within lung parenchyma of BALB/c mice while in CBA/Ca it accumulates within peribronchial and perivascular areas [3], [9]. Fibroblasts are activated during tissue injury and they can also recognize pathogen-associated molecular patterns (PAMPs) [47]. Rapid activation of TGFβ by parenchymal fibroblasts could explain observed TGFβ-mediated increase in Treg population, a feature of BALB/c infection [21]. Considering all the evidence it can be easily envisaged that the differences in the fibroblast phenotypes contributed to the observed differences in the immune cell migration, TGFβ availability and subsequently to the susceptibility to pneumococcal infection.

The large accumulation of immune cells within perivascular areas of CBA/Ca but not BALB/c mice suggests that the vasculature is involved in the resistance. We showed that perivascular areas of resistant BALB/c mice were either unaffected or mildly inflamed, even when massive inflammation within lung parenchyma was observed. The accumulation of leukocytes within perivascular areas could cause damage to the vessel structure and facilitate bacterial dissemination into the blood, which is a feature of infection of CBA/Ca but not of BALB/c mice. The hypothesis that alteration of the vasculature is a key aspect of susceptibility is further supported by analysis of our pulmonary gene expression data that showed one of the most enriched functional gene clusters during the pneumococcal infection is for the remodelling of lung vasculature. Furthermore, we have found a group of candidate genes with identified phenotype-associated polymorphism to be involved in smooth muscle functioning (*Ryr1*, *Kcnk6*, *Ntrk2*, *Ppp1r14a* and *Kif20b*). *Ryr1* is a calcium channel [57], [58], *Kcnk6* is a potassium channel [59], *Ppp1r14p* is a phosphatase inhibitor [60], *Ntrk2* is a neutrophin receptor [61], [62] and *Kif20b* encodes a kinesin [63]. The *Ntrk2* knock-out mice show significantly reduced blood vessel formation [64] and *Kcnk6* knock-out mice show vascular dysfunction [59]. Smooth muscles are a product of fibroblast differentiation and an important component of airways and lung vasculature [65], [66], [67].

Interestingly, we found that the enrichment of genes belonging to the adipocytokine signalling pathway was unique for the CBA/Ca pulmonary transcriptome. This is worth noting because the adipocytokines are secreted predominantly by adipose tissue, which in the lung is located within perivascular areas. This perivascular adipose tissue (PVAT) plays an important role in the regulation of vascular structure and homeostasis [65]. It is known that leukocyte infiltration and their oxygen radical production are key factors in PVAT dysfunction, which in turn leads to compromised blood vessel functionality [68]. Thus the accumulation of immune cells within perivascular areas of CBA/Ca mice is likely to be affecting PVAT functioning. Consistent with this, we observed that the expression of resistin-like molecule γ (*Retnlg*), which is secreted by adipocytes, was almost 3-fold higher in the susceptible CBA/Ca mice during pneumococcal infection.

PVAT is also an important element of a brain-blood vessel axis because nerve endings are present in the PVAT, enabling neuronal control of vessel function [69]. It is therefore of interest that the genes involved in axon guidance were significantly over-represented in the pulmonary transcriptome of infected BALB/c mice. We also found that the axon guidance receptor gene *Dcc* (netrin receptor Dcc) was encoded within the QTL on chromosome 18, in direct proximity of the cluster of highly significant intergenic SNPs. In addition to the axon guidance pathway being involved in the formation of the neuronal network that can control blood vessels and bronchial contractility, it can also regulate leukocyte transmigration from blood to lung tissue [70], [71]. This may offer a further explanation to better clearance of immune cells from the perivascular areas of infected BALB/c mice.

It is noteworthy though, that a candidate gene *Ntrk2* (in the QTL on chromosome 13), for a neutrophin receptor, is also involved in cross-talk between nervous and immune systems [72], regulation of Th1/Th2 polarisation [73], T-cells development [74] and smooth muscle cells proliferation [75]. This wide range of *Ntrk2* functions places this gene in a central position linking airway smooth muscle, nervous and immune system, three areas where differences were found between resistant and susceptible strains during pneumococcal infection. In this context it is also notable that *Ntrk2* was recently described as a protecting factor in pneumococcal meningitis [76], making this gene a very promising candidate for further investigation.

There is clear evidence that regulation of immune cell populations differs between resistant and susceptible strains during pneumococcal infection. This can be seen in timing and quantity of cellular infiltrations [3], [9], T-cell recruitment [21] and macrophage functioning [77]. Furthermore, the results from our present study showed that pathways that differentiated between pulmonary transcriptomes of resistant and susceptible mice were associated with immune cells proliferation and functioning. We showed that hematopoietic cell lineage and p53-signalling pathways are unique for BALB/c, while pathways involved in immune cells functioning such as T-cells receptor signalling and natural killer cells mediated cytotoxicity were unique to susceptible CBA/Ca mice. Furthermore, we found that candidate genes *Fas*, *Map4k1*, *RasGRP4* and *Spint2* (all but *Fas* encoded within the QTL on chromosome 7) were involved in regulation of immune cells. For example, *RasGRP4* encodes guanine nucleotides exchange factor, which was recently reported to influence ROS production in neutrophils [78] and *Spint2* encodes a Kunitz-type serine protease inhibitor. Interestingly, a Kunitz-type protease inhibitor was reported to suppress pro-inflammatory cytokine production in macrophages and neutrophils [79], [80], [81] offering possible explanation for observed differences in BALB/c and CBA/Ca macrophages [77]. Two very interesting genes were *Fas* and *Map4k1* both of which showed phenotype-associated amino acid substitutions. Their expression also differed between CBA/Ca and BALB/c in disease or between control animals. Although *Fas* regulates apoptosis in a wide range of cells, *Map4k1* is expressed exclusively in hematopoietic compartment. *Map4k1* plays a pivotal role in regulation of activation induced T-cell apoptosis [82]. This function seems especially interesting because many reports demonstrate that T-cells are recruited and highly engaged during pneumococcal infection [8], [21], [83] and affect host survival [21], [84], [85].

The regulation of immune cell populations is a key factor in resistance to pneumococci that can lead to either resolution (associated with resistance) or uncontrolled inflammation (associated with susceptibility). The trafficking of immune cells from the bloodstream to tissues parenchyma is regulated by fibroblasts and may be critical for the host early defences against pneumococci [51]. Furthermore our data demonstrate that lung vasculature may be equally important to host survival, not least because it constitutes an important barrier to bacterial dissemination into blood and development of invasive pneumococcal disease. The accumulation of immune cells within lung perivascular areas could affect vessel functioning and facilitate bacterial dissemination into blood, a prominent feature of susceptible mice infection. In contrast, migration of leukocytes into lung parenchyma could activate parenchymal fibroblasts and initiate TGFβ-mediated immunomodulatory responses, a feature of resistant mice infection. This pattern of inflammation is likely a result of inherited differences in lung vasculature (e.g.: fibroblast/smooth muscle cells) and/or immune cells qualities (e.g.: motility or ability to clear bacteria). This is a promising hypothesis since we found that half of the genes carrying phenotype-associated polymorphism have well documented roles in fibroblast differentiation, smooth muscle and immune cells functioning. Therefore we propose that the phenotype of pulmonary fibroblasts may contribute to host survival through regulation of immune cells trafficking, synthesis and activation of TGFβ and modulation of vessel functioning. Furthermore we speculate that the resistance to pneumococcal infection is modulated by the cumulative effect of alteration within several genetic factors. This hypothesis comes from the discovery of multiple disease QTLs and observation that the phenotype-associated polymorphism was located within very limited number of genes and intergenic regions (22 genes out of few hundred encoded within identified four QTLs). We also observed mixed pattern of susceptible and resistant SNP variants associated with intermediate but not susceptible mouse strains, supporting our speculation on the cumulative effect of candidate genes on resistance to pneumococci.

## Methods

### Ethics statement

All animal experiments were performed in accordance to the UK Home Office guidelines. The University of Leicester Ethics Committee and the UK Home Office approved the study protocols. Animals were housed and experiments carried out at designated facility at the University of Leicester.

### Mice

The female mice of 26 inbred Jax-mouse strains, 10 mice per strain were purchased from The Jackson Laboratory: 129S1/SvImJ, A/J, AKR/J, BALB/cByJ, BALB/cJ, BTBR T+ tf/J, C3H/HeJ, C57BL/J, C57BL/6J, C57BL/10J, C57BLKS/J, C57BR/cdJ, C58/J, CBA/J, DBA/1J, DBA/2J, FVB/NJ, I/LnJ, LP/J, MA/MyJ, NOD/ShiLtJ, NZW/LacJ, PL/J, SEA/GnJ, SJL/J and SM/J while two strains BALB/cOlaHsd and CBA/CaOlaHsd were purchased from Harlan, UK. Mice were housed in the Division of Biomedical Services, University of Leicester. All mice were fed on fundamental diet. Mice used for the experiments were 9 to 12 week old.

### Bacterial strain

*Streptococcus pneumonia* serotype 2 strain D39 (NCTC7466) from the National Collection of Type Cultures, Central Public Health Laboratory, London, United Kingdom was used. To prepare a challenge stock bacteria were grown overnight on blood agar base (BAB) plate with 5% horse blood in CO_2_ jar at 37°C. Next day bacterial colonies were transferred from the BAB plate to THY media (Gibco) until OD_600_ reached 0.2. The primary culture was diluted 100 times in THY and grown at 37°C in anaerobic condition until the culture reached OD_600_ corresponding to 1–3×10^8^ CFU/ml (middle exponential phase). At this point culture was mixed with glycerol (final concentration 10% v/v), aliquot and frozen at −70°C. Culture was stored up to 3 months.

### Infection

To prepare the challenge dose, aliquot of bacteria stock was centrifuged (13 K rpm) and pellet resuspended in PBS to final concentration 2.5×10^8^ CFU/ml. Mice were lightly anaesthetized with 2.5 to 5.0% (v/v) fluothane (Zeneca Pharmaceuticals, Macclesfield, United Kingdom) over oxygen (1.5 to 2 l/min) and intranasally infected with 20 µl (5×10^6^) of the challenge dose. 24 h post-infection about 50 µl of blood were taken from the tail vain and bacteria load in blood was measured by viable count (Gingles at al 2001). Mice were closely monitored and the pain score chart was filled every 6 h until animal was lethargic or until the end of the experiment (7 days) when animal was classified as a survivor. This time point was then used as a surrogate for the survival end point. Animals were humanly euthanized by cervical dislocation when they reached the end point, either when lethargic or at the end of the experiment (168 h). At the time of death blood and lung were collected and bacterial load was measured by viable count.

### Phenotype analysis

The time the animal survived the infection (survival), was used for genome-wide associations mapping to identify disease QTLs. Collected data for each mouse were first analysed using statistical software SPSS. Bartlett's test for equal variances was calculated for each mouse strain and phenotype. One-way ANOVA and Tukey's Multiple Comparison post-test were used to check for statistically significant differences between strains. Strains were classified as resistant, sensitive or intermediate based on the ANOVA results of the survival time for each strain. The rate of survival was represented as the percentage of animals that survived the infection (168 h).

### Microarray

Pulmonary gene expression in CBA/CaOlaHsd and BALB/cOlaHsd mice (Harlan, UK) was assessed at 6 h post infection. Mice inoculated with 20 µl of PBS and sacrificed 6 h post-inoculation were treated as control. Six control animals and five infected animals per strain were used in the gene expression experiment. Control and infected mice were sacrificed at 6 h time point and whole lung was removed. Lungs were cut into small squares and immediately immersed in RNAlater (Qiagen). Lung RNA was isolated using RNAeasy mini kit (Qiagen) according to manufacturer instructions. cRNA was synthesized using Ambion MessageAmp kit for Illumina arrays according to manufacturer instruction. The whole lung gene expression was done using chip MouseWG-6_V2_0_R3 on Illumina platform. Gene expression data deposited within the NCBI Gene Expression Omnibus (GEO), Accession No: GSE49533.

### Analysis of microarray data

Microarray data were analysed using GenomeStudio (Illumina) and ArrayTrack [86]. Signal intensity data were normalized across all arrays using quintile normalization in GenomeStudio (Illumina) software. The lists of differentially expressed genes were then created using t-test in ArrayTrack. Differentially expressed genes were selected by calculating a ratio between signal intensity of infected and non-infected conditions for each mouse strain or by calculating the ratio between signal intensity of control BALB/cOlaHsd and CBA/CaOlaHsd animals to check for background differences unrelated to infection.

### Real-time PCR

For quantitative RT-PCR, 500 ng of total RNA was reverse transcribed using the RevertAid H Minus First Strand cDNASyntheis Kit according to manufacturer protocol (Fermentas). Real time quantitative PCR was performed in triplicate on a LightCycler 480 Real-Time PCR System (Roche) in a final volume of 5 µl using 1× Maxima SYBR Green qPCR master mix (Fermentas) and 3.3 µM of forward and reverse primers for each mRNA tested. Cycling conditions were 95°C for 15 minutes, and 38 cycles of amplification at 95°C for 15 seconds, 60°C for 30 seconds and 72°C for 30 seconds. Primers were designed to amplify the same transcripts as those tested on the Illumina MouseWG-6_V2_0_R3 whole genome gene expression beadchips (Table S3). Gapdh and Hprt-1 mRNAs were selected for data normalisation using Genorm [87]. The expression of each mRNA tested was normalised to the geometric mean of Gapdh and Hprt-1 expression [87].

### Genome Wide Association Mapping by PLINK

Genotype files (130K SNP panel, Mouse Assembly NCBIM37) for phenotyped inbred Jax-mouse strains were downloaded from Mouse Phenome Database (MPD). To search for haplotype associations (Haplotype Association Mapping, HAM) the mean survival time (hours) was used. Data were analysed using gPLINK version 2.050 [88].

### Genome Wide Association Mapping by EMMA

Measurements for survival in hours for 10 animals per mouse strain were used to search for association using Efficient Mixed Model Association, EMMA [89]. Analyses were done using EMMA Correction Server for all tested inbred mouse strains. EMMA algorithm corrects for population structure and relatedness among inbred mouse strains. QTL mapping by EMMA was done using both 130K and 4Mil SNP panels available.

### QTL selection

The QTLs were consider a candidate disease QTL if at least 3 SNPs within 0.5 Mb were found displaying significance equal or below p-value 10^−5^ for 130K SNP panel and p-value equal or below 5×10^−8^ when 4Mil SNP panel was used. The interval of the QTL was calculated as the region containing significant SNPs with 1 Mb downstream and upstream of this region [90]. The list of candidate QTLs was generated and cross-compared between both methods. QTL that was significant in both EMMA and PLINK was selected as candidate QTL.

### Sequence polymorphism within Harlan BALB/c and CBA/Ca strains within candidate QTLs

For sequencing of BALB/cOlaHsd and CBA/CaOlaHsd mice, DNA was extracted from mouse-tail tissue using the Nucleon kit (Gen-probe). Sequencing was performed at the Wellcome Trust Centre for Human Genetic's High Throughput Genomics Facility (UK), generating 8- to 10-fold coverage of a single lane of PE100 nt per sample on the HiSeq2000. Paired-end Illumina reads from CBA/CaOlaHsd were aligned to the reference genome (C57BL/6J, NCBIM37) using BWA, the same was done for BALB/cOlaHsd. Detection of SNPs in each alignment was made using the Genome Analysis Toolkit (GATK) with default parameters. We adopted a filtering strategy to identify high-confidence SNPs and reduce false positives. SNP sites that failed GATK's internal status check, quality <300 or had an allele ratio <0.8 were removed from any further analysis. Differing SNPs between CBA/CaOlaHsd and BALB/cOlaHsd were then compared to the precompiled SNPs found in 11 strains from the Mouse Genome Project (129S1/SvImJ, A/J, BALB/cJ, C3H/HeJ, CBA/J, FVB/NJ, AKR/J, NOD/ShiLtJ, DBA/2J, C57BL/6J and LP/J). We generated a list of SNPs between CBA/CaOlaHsd and BALB/cOlaHsd that were copied in CBA/J and BALB/cJ; this was used as the final list of differentiating SNPs. List of SNPs was deposited in NCBI dbSNP [91].

### Gene Identification

Gene list were generated for each selected QTL using MGI Genes and Markers Query Form. Genes within selected QTLs were then compared with the lists of differentially expressed genes during pneumococcal infection of BALB/c and CBA/Ca strains (ArrayTrack). The list of differentially expressed genes between non-disease condition in BALB/cOlaHsd and CBA/CaOlaHsd was also analysed. The sequence of the genes within QTL region was examined using Mouse Phenome Database (MPD) SNP variation query form (NCBIM37). The gene was classified as most likely causal candidate if found within susceptibility QTL and either showed genetic polymorphism within or nearby its coding region or showed change in expression during pneumococcal infection. The identified nucleotide substitutions within candidate genes that led to amino acid change were also analysed using the PROVEAN tool [92] to evaluate an effect of the alteration on the protein function.

### Preparation and analysis of lung tissues sections

At necropsy, tissue samples were immersed in 10% v/v neutral buffered formaldehyde solution prior to conventional processing and embedding in paraffin wax. Histopathological assessment was performed on tissue section stained with haematoxylin and eosin (BDH). Lungs samples were then examined microscopically and vessels with visible perivascular areas were counted as affected (accumulation of cellular influx) or unaffected (clear perivascular areas). The number of affected vessels per lung was then calculated as a percentage of total number of counted vessels (affected and unaffected) in the lung section. Lungs of five animals per time point (6 h and 24 h) and per mouse strain were examined.

## Supporting Information

Table S1 **The genome-wide significant SNPs (p-value<5×10E-8) identified during the EMMA mapping and their alteration within tested inbred mouse strains.** The SNP ID, Ensemble annotation for the SNP (I – intron variant, U3 – UTR variant 3′, Cn – missense, Cs synonymous codon) and log10 p-value scores for the EMMA mapping [GWAS log(p)] are presented. The red colour indicates resistant mouse strain or SNP associated with the resistance to pneumococcal infection.
(XLSX)

Table S2 **Correlation between expression scores obtained by microarray and RT-qPCR technique.**
(XLSX)

Table S3 **Primer pairs used for RT-qPCR of selected mouse genes.**
(XLSX)
